# Recent Advancements Surrounding the Role of the Periaqueductal Gray in Predators and Prey

**DOI:** 10.3389/fnbeh.2019.00060

**Published:** 2019-05-10

**Authors:** Tamara B. Franklin

**Affiliations:** The Social Lab, Department of Psychology and Neuroscience, Dalhousie University, Halifax, NS, Canada

**Keywords:** periaqueductal gray, hunting, defensive behavior, freezing, escape, fear

## Abstract

Recent advances in neural circuitry techniques, like optogenetics and chemogenetics, have allowed for a greater understanding of the periaqueductal gray (PAG) and its importance in predator and prey behaviors. These studies in rodents have highlighted the role of the rostrolateral PAG in hunting behaviors, and have demonstrated functional differences across the dorsal-ventral/rostral-caudal axes of the PAG associated with defensive behaviors. Human imaging studies have further demonstrated that the PAG is active during situations involving imminent threat suggesting that the function of the PAG is likely largely conserved across species. This mini-review article highlights some of the recent advancements towards our understanding of the functional neuroanatomy of the PAG and its importance in the predator and prey behaviors that are critical for survival.

The periaqueductal gray (PAG) is essential for the expression of both the hunting behaviors performed by predators and the defensive behaviors performed by prey. Anatomically, it is largely bordered dorsally by the superior colliculus, and ventrally by the dorsal raphe (DR) and midbrain reticular nucleus. It can be further sub-divided into four columns arranged around the cerebral aqueduct (dorsomedial, dorsolateral, lateral, ventrolateral; Figure 1). These columns can be identified in part by their cytoarchitecture, by the presence of nicotinamide adenine dinucleotide phosphate (NADPH)-positive neurons in the dorsolateral PAG, and by distinct afferent and efferent connections (Vianna and Brandão, 2003). The PAG is sometimes defined in functional terms within the context of its dorsal (including dorsolateral and dorsomedial columns) and ventral (including lateral and ventrolateral columns) regions.

**Figure 1:**
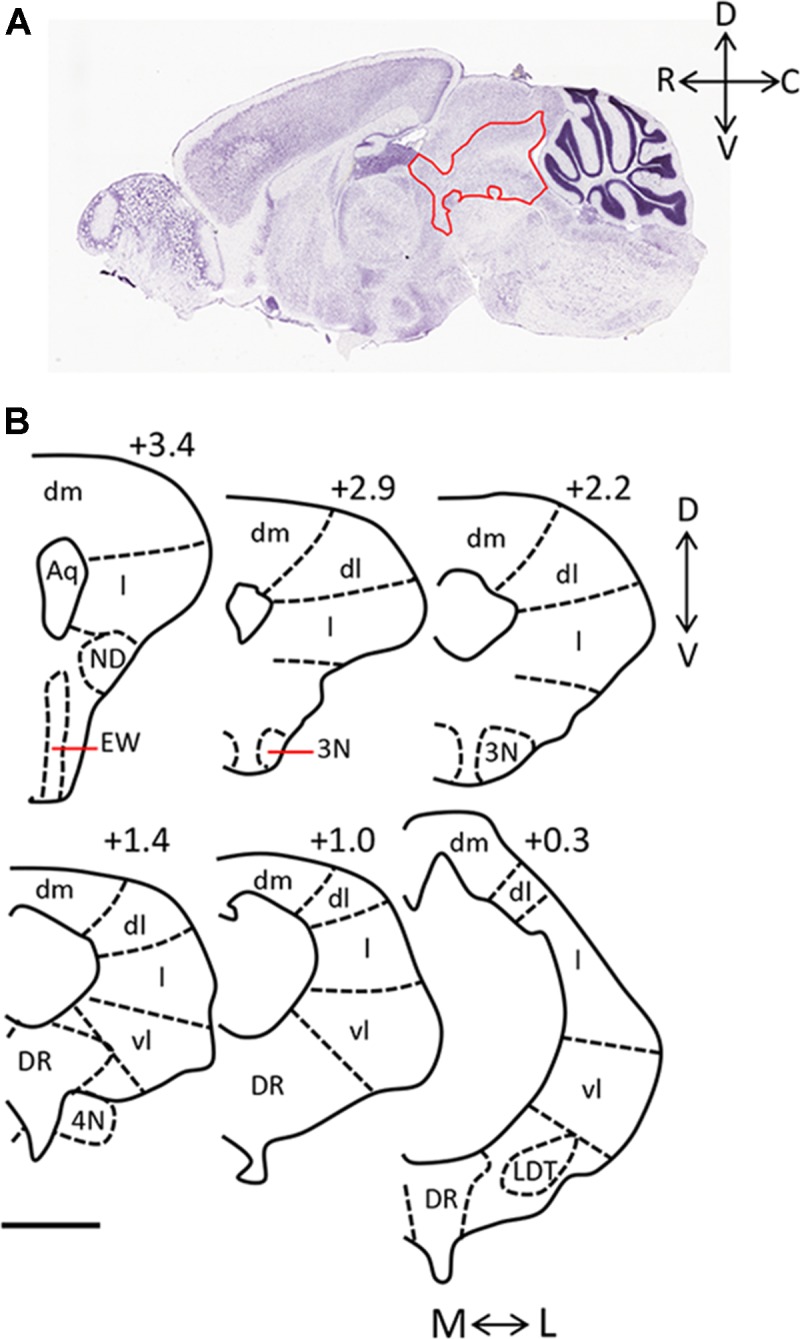
Anatomy of the periaqueductal gray (PAG) in the rodent. **(A)** Sagittal section of mouse brain (Image credit: Allen Mouse Brain Nissl Sagittal Atlas, Image 21, 2004). The PAG is outlined in red and rostral (R), caudal (C), dorsal (D), and ventral (V) axes are indicated. **(B)** Camera lucida drawings of coronal sections taken through the rat PAG, with the dorsomedial (dm), dorsolateral (dl), lateral (l) and ventrolateral (vl) columns labeled (adapted from Comoli et al., 2003, with permission from Elsevier). Location of each section relative to the interaural line is included (+3.4, +2.9, +2.2, +1.4, +1.0, 0.3), and the medial (M)—lateral (L), and D-V axes are indicated. Scale bar, 1 mm. Abbreviations: 3N, oculomotor nucleus; 4N, trochlear nucleus; Aq, aqueduct; dl, dorsolateral; dm, dorsomedial; DR, dorsal raphe; EW, Edinger-Westphal nucleus; l, lateral; LDT, laterodorsal tegmental nucleus; vl, ventrolateral.

The PAG recognizes and generates appropriate behaviors in response to a variety of aversive stimuli including pain and hypoxia (Lau and Vaughan, 2014; Schenberg et al., 2014) but the focus of this mini-review is its role in predation and prey behaviors. While it is clear that the PAG is critical in producing behaviors in the hunting and the hunted, the involvement of different subareas differs depending on the behavior expressed. For example, in the rat, exposure to a cat results in the highest expression of Fos, an immediate early gene used as a marker of cell activation, in the rostral dorsomedial and dorsolateral PAG, and in the caudal lateral and ventrolateral PAG (Canteras and Goto, 1999; Comoli et al., 2003). This differs from the neural activation resulting from insect predation, where Fos expression is highest in the rostrolateral PAG (Comoli et al., 2003). This mini-review integrates recent circuit-based studies to discuss the role of different sub-areas of the PAG in the predator and prey behaviors which are essential to survival.

## The Role of the PAG in Predatory Hunting

The rostrolateral PAG, most often defined at the level of the oculomotor nucleus, has emerged as an area that is critical for predatory behaviors in cats, rats and mice (Shaikh et al., 1987; Sukikara et al., 2006; Mota-Ortiz et al., 2012; Tulogdi et al., 2015). Lesions of the rostrolateral PAG in male rats greatly reduce the incidents of attacking or chasing insect prey (Mota-Ortiz et al., 2012). Furthermore, the rostrolateral PAG plays a critical role in the switch from maternal behaviors to predatory hunting which occurs in morphine-sensitized dams that have been further exposed to low doses of morphine (Sukikara et al., 2006). Morphine-treated dams will ignore their pups, and instead will increase their predatory behavior towards insects, but morphine-treated dams with lesions of the rostrolateral PAG perform reduced insect hunting and more maternal care. This suggests that the rostrolateral PAG is an important part of an opioid-dependent, potentially adaptive response, which can be recruited in times when food sources are limited. The rostrolateral PAG has also been implicated in predatory aggression, unrelated to a food source, in experiments comparing neural activation in muricidal rats with non-muricidal rats (Tulogdi et al., 2015). Rats who have committed muricide display a shift in brain activation towards ventral areas compared to dorsal areas of the PAG, and this occurs, particularly in the rostral and not caudal areas. Overall, these findings are part of a growing number of studies describing the PAG as a brain area able to integrate information concerning different motivational states to express appropriate adaptive behaviors.

Studies suggest that the role of the PAG in predatory behavior may be associated with a more general role for the PAG in reward processing. A recent study using a GABA_A_ agonist to inactivate the rostral lateral and dorsolateral columns of the PAG demonstrated that inhibition of this region reduces food consumption and that this may be due to reward-responsive neurons in the lateral or dorsolateral PAG (Tryon and Mizumori, 2018). Further, evidence for the role of the PAG in reward processing comes from a study focused on the efferent projections from the rostrolateral PAG to orexinergic neurons in the lateral hypothalamus which are thought to be critical in determining motivation to chase prey (Mota-Ortiz et al., 2012); orexinergic neurons have been reported to be important for reward processing in general and are associated with the rewarding aspects of both food and drugs (Harris and Aston-Jones, 2006). Whether the PAG plays a significant role in reward processing that is contributing to its regulation of predatory drive has yet to be determined.

Projections to the PAG, that could be mediating the predator behaviors described, include substantial projections from areas in the prefrontal cortex, amygdala, and hypothalamus (Floyd et al., 2000; Han et al., 2017; Li et al., 2018; Park et al., 2018). Although, these projections do not target the rostrolateral regions of the PAG specifically, optogenetic and chemogenetic experiments identifying pathways required for predatory behaviors have identified projections to and from the ventral PAG as being critical. The central nucleus of the amygdala mainly has inhibitory projections to the ventral PAG, and activation of this pathway leads to enhanced prey pursuit (Han et al., 2017). Similar to projections from the amygdala, the projection from the lateral hypothalamus to the ventral PAG is also made up of mainly GABAergic neurons and it is these neurons that drive predatory attack (Li et al., 2018). Interestingly, stimulation of excitatory, rather than inhibitory, afferent connections leading from another hypothalamic area, the medial preoptic area, to the ventral PAG increases hunting behaviors towards both an inanimate object and natural prey in mice (Park et al., 2018). This suggests that the glutamatergic projections from the medial preoptic area, and the GABAergic projections from the central amygdala and lateral hypothalamus, may target different cell types within the microcircuitry of the PAG such that both excitatory and inhibitory input ultimately leads to a coordinated behavioral output.

## The Role of the PAG in Prey

Prey rely on a range of behavioral responses to increase the likelihood of survival in the face of dangerous and life-threatening situations. These include active fight/flight responses and passive freezing behaviors, which can be either innate or learned (conditioned). Environmental factors, like the presence of escape routes, and the proximity to the threat, contribute to the type of defensive behavior elicited. In the case of distal threats, rodents will engage in freezing behaviors, and will switch their defensive response to escape-related behaviors like flight and jumps as the likelihood of attack increases (Bolles and Fanselow, 1980). Interestingly, this diverse range of complex behavioral responses are all mediated, in part, by the PAG. Perhaps not unexpectedly, there is widespread activation in the PAG, as measured by c-fos, after exposure to a predator odor (Dielenberg et al., 2001). Evidence for region-specific functional roles related to the varied and sometimes opposite behavioral responses are discussed below.

### Evidence for a Dorsal-Ventral Functional Division

Common understanding of PAG function in defensive behaviors is that the dorsal and ventral PAG have two apparently opposing actions on behavior; traditionally, dorsal PAG activity is largely associated with escape and flight, while ventral PAG activity is responsible for freezing behaviors (Adamec et al., 2012; Assareh et al., 2016; Silva et al., 2016; Tovote et al., 2016; Watson et al., 2016; Vieira-Rasteli et al., 2018). However, recent studies using single-unit recordings and optogenetic/chemogenetic techniques have painted a more complex picture.

Overall, neurons in the dorsal PAG increase their firing rate more in response to exposure to a predator odor than neurons in the ventral PAG (Watson et al., 2016). In support of the theory that the ventral PAG generates freezing behaviors, optogenetic manipulations have demonstrated that an inhibitory pathway leading from the central nucleus of the amygdala to GABAergic neurons in the ventrolateral PAG acts to disinhibit glutamatergic outputs to the magnocellular nucleus of the medulla; this pathway is critical for producing learned freezing behaviors in response to a footshock (Tovote et al., 2016). This projection appears to be independent of the projection from the central nucleus of the amygdala to the PAG associated with hunting behavior. Glutamatergic neurons in the ventrolateral PAG are also required for the animal to express normal freezing behaviors when exposed to a moving object that visually mimics a predator, highlighting these output neurons as important mediators of freezing behaviors (Tovote et al., 2016).

Interestingly, optogenetic activation of GABAergic neurons in the ventrolateral PAG, or the glutamatergic neurons in the dorsal PAG which project to these neurons, result in flight; this neural microcircuitry likely facilitates the rapid switch between active and passive defensive responses observed in rodents who perceive imminent danger (Tovote et al., 2016). Findings that optogenetic activation of glutamatergic projections from the lateral hypothalamus to the ventral PAG result in escape behaviors (fleeing and jumping) from a moving object (Li et al., 2018) suggest that neurons in the hypothalamus may be acting *via* their actions on GABAergic neurons in the ventral PAG or glutamatergic neurons in the lateral PAG, both of which receive similar amounts of projections from the lateral hypothalamus (Tovote et al., 2016).

In support of the theory that the dorsal PAG is responsible for escape behaviors, a recent study using optogenetics and calcium imaging has demonstrated that glutamatergic neurons in the dorsal PAG encode the decision to escape an aversive stimulus and the speed at which this escape occurs (Evans et al., 2018). However, in addition to flight, the dorsal PAG has the ability to also control risk assessment and freezing (Vianna and Brandão, 2003; Bittencourt et al., 2004; Aguiar and Guimarães, 2009; Assareh et al., 2016; Deng et al., 2016). Whether stimulation of the dorsal PAG results in freezing or escape behaviors appears to depend on the strength of the stimulus; higher levels of electrical current applied to the dorsal PAG (Vianna and Brandão, 2003; Bittencourt et al., 2004) and in the lateral PAG (Assareh et al., 2016) resulting in escape behaviors. Interestingly, cell-type specific activation of excitatory neurons in the dorsal PAG can mediate both these diverse responses; optogenetic stimulation of CamKIIα-positive neurons in the dorsal PAG results in both increased flight and freezing (Deng et al., 2016). Single-unit *in vivo* electrophysiology performed during exposure of a mouse to a predator (rat) identified distinct subsets of dorsal PAG neurons that are responsible for risk assessment, flight, and freezing with a very small percentage of cells firing in association with more than one of these behaviors (Deng et al., 2016).

Acute predator exposure in rodents is used as a model of post-traumatic stress disorder because of its long-term and persistent effects. The long-term neural plasticity induced by a single exposure to a predator observed in the PAG differs according to dorsal and ventral subdivisions. Phosphorylated cAMP response element binding protein (pCREB) is a protein that regulates expression of many synaptic plasticity-related genes, and thus its expression can be used as a marker of neural plasticity. pCREB expression is increased transiently 20 min following predator exposure in the lateral PAG (Adamec et al., 2003, 2009) but this is associated with a potentiation in transmission from the central nucleus of the amygdala to the lateral PAG (Adamec et al., 2003). Artificially increasing pCREB expression in non-predator exposed rats in the lateral PAG can also increase potentiation from the central nucleus of the amygdala to the lateral PAG and is anxiogenic mimicking what is observed in predator exposed rats (Adamec et al., 2009). pCREB expression is also persistently increased in the dorsal PAG, and decreased in the ventral PAG, 1 and 7 days after predator exposure (Adamec et al., 2011). While this data supports a role for the PAG in fear memory formation resulting from predator exposure, this is controversial. Experiments involving chemogenetic silencing of the dorsal PAG in mice suggested that the dorsal PAG is needed for the expression of acute fear behaviors on exposure to a predator, but that this is not required for the formation of the fear memory; mice are able to show learned fear to the context in which the predator exposure took place despite not showing an acute fear response at the time of predator exposure (Silva et al., 2016).

### Evidence for a Rostral-Caudal Functional Division

While many studies investigating the role of the PAG in rodent defensive behaviors consider columns of the PAG, less focus has been placed on differences that may occur from rostral to caudal ends, and few studies have directly compared the function of rostral and caudal regions. However, the studies that have investigated rostral-caudal position as a factor have suggested key differences in function across the rostral-caudal axis. Cat-induced activation of the rat PAG, as measured by Fos expression, suggests that a distinct rostral-caudal gradient occurs in combination with a dorsal-ventral gradient (Canteras and Goto, 1999; Comoli et al., 2003). This differing activation across a rostral-caudal axis has also been observed at a causal level in experiments using local infusions of an NMDA antagonist, AP5, to block activity in the rostral or caudal dorsolateral PAG (Souza and Carobrez, 2016). Excitatory transmission *via* NMDA receptor activation in the rostral dorsolateral PAG is associated with the expression of innate defensive responses to predator odor and the subsequent expression of fear to the context previously paired with the odor. Souza and Carobrez additionally described that, while excitatory transmission in the rostral dorsolateral PAG is not required for consolidation of predator odor-induced contextual fear, NMDA-mediated activity in the caudal dorsolateral PAG is required to maintain contextual fear conditioning (Souza and Carobrez, 2016). These findings differ from Silva et al. (2016), described above, which suggested that the dorsal PAG is associated predominantly with the motor output of the fear response rather than formation of the contextual fear memory itself. The difference between these two findings may highlight the potential importance of the fear stimulus itself; Souza and Carobrez (2016) used a predator odor while Silva et al. (2016) used a live rat raising the possibility that engaging multiple sensory modalities, rather than solely olfaction, recruits several pathways that can result in formation of a fear memory independent of the dorsal PAG. This again underlines the complexity, as well as the controversy, surrounding the role of the PAG in memory. In addition, to this point, a rostral-caudal functional differentiation has focused on the dorsal PAG; further work is required to better understand whether rostral and caudal, lateral and ventrolateral areas are also responsible for differing aspects of innate and learned fear responses associated with predator exposure.

### Human PAG Activation Associated With Fear of Predators

Human imaging studies have clearly demonstrated that the PAG is activated when humans are exposed to stimuli that suggest danger. Blood-oxygen-level dependent (BOLD) activity in the PAG increases as innately threatening stimuli (i.e., a virtual predator or a tarantula) becomes more imminent (Mobbs et al., 2007, 2009, 2010; Coker-Appiah et al., 2013). BOLD responses in the PAG are also increased on exposure to negatively valenced, aversive pictures and this is correlated with fear bradycardia (heart rate deceleration), the result of activation of the parasympathetic nervous system, akin to freezing behavior in rodents (Hermans et al., 2013). Activity in the PAG resulting from exposure to aversive pictures is also correlated with increased activity in the amygdala suggesting that the amygdala-PAG pathway is involved in the processing of aversive stimuli (Hermans et al., 2013). This pathway has similarly been shown to be important in the processing of threat (Mobbs et al., 2009) further paralleling studies on the amygdala-PAG pathway described in rodents above.

Perhaps due to the limited spatial resolution of functional magnetic resonance imaging (fMRI) technology, much of this research has not sub-divided the human PAG into its dorsal and ventral sub-areas (Linnman et al., 2012). However, one study investigating resting-state functional connectivity in the dorsolateral and ventrolateral PAG of post-traumatic stress disorder patients does suggest that a functional subdivision associated with active vs. passive coping is present in humans, similar to that observed in animals (Harricharan et al., 2016). Overall, these studies in humans suggest that the function of PAG recruitment on exposure to a threat may be largely conserved between rodents and humans thus strengthening the utility of rodent studies aimed at understanding the complex role that the PAG plays in prey behaviors.

## Author Contributions

TF is the sole author of this review article.

## Conflict of Interest Statement

The author declares that the research was conducted in the absence of any commercial or financial relationships that could be construed as a potential conflict of interest.
